# An evaluation of techniques to diagnose *Dioctophyme renale* in dogs

**DOI:** 10.29374/2527-2179.bjvm006423

**Published:** 2024-02-05

**Authors:** Gabriela de Almeida Capella, Josaine Cristina da Silva Rappeti, Natalia Berne Pinheiro, Soliane Carra Perera, Micaele Quintana de Moura, Marlete Brum Cleff, Caroline Maciel da Costa, Adriane Leites Strothmann, Guilherme Borges Weege, Carolina Silveira Mascarenhas, Maria Elisabeth Aires Berne

**Affiliations:** 1 Veterinarian, DSc, Programa de Pós-Graduação em Microbiologia e Parasitologia, Instituto de Biologia, Universidade Federal de Pelotas (UFPEL), Pelotas, RS, Brazil.; 2 Veterinarian, DSc, Departamento de Clínicas Veterinária, Faculdade de Veterinária, UFPEL, Pelotas, RS, Brazil.; 3 Veterinarian,DSc, Instituto de Biologia, UFPEL, Pelotas, RS, Brazil.; 4 Veterinarian, DSc, Programa de Pós-Graduação em Veterinária, Departamento de Clínicas Veterinária, Faculdade de Veterinária, UFPEL, Pelotas, RS, Brazil.; 5 Biologist, DSc, Programa de Pós-Graduação em Microbiologia e Parasitologia, Instituto de Biologia, Universidade Federal de Pelotas (UFPEL), Pelotas, RS, Brazil.; 6 Undergraduate in Zootechnics, UFPEL, Pelotas, RS, Brazil.; 7 Undergraduate in Biologic Sciences, UFPEL, Pelotas, RS, Brazil.; 8 Biomedic, DSc, Programa de Pós-Graduação em Microbiologia e Parasitologia, Instituto de Biologia,UFPEL, Pelotas, RS, Brazil.; 9 Biologist, DSc, Instituto Federal Sul-rio-grandense (IFSul), Campus Pelotas, RS, Brazil.

**Keywords:** Indirect ELISA, ultrasound, urinalysis, dioctophimosis, ELISA Indireto, ultrassom, urinálise, dioctofimose

## Abstract

*Dioctophyme renale* is a nematode with zoonotic potential that affects the kidneys of carnivorous, wild, and domestic mammals. In this study, we sought to evaluate the indirect ELISA method against routine methods used to diagnose dioctophimosis. Hence, 38 dogs parasitized by *D. renale*, as confirmed by surgery, were selected. The dogs were evaluated by abdominal ultrasound and urinalysis, and their sera were tested by indirect ELISA using *D. renale* adult secretion and excretion antigen (DES). Five dogs were followed up with serum collections on day 0 (day of surgery) and 30, 60, and 90 days after surgery to evaluate antibody kinetics. Abdominal ultrasound and indirect ELISA successfully diagnosed 37 dogs parasitized by *D. renale*, while urinalysis diagnosed 29 animals. The positive animals were parasitized with 1-7 parasites; 17 dogs were infected by male and female parasites, 15 only by female parasites, and six were parasitized only by male parasites. When assessing specificity and sensitivity, all techniques showed 100% specificity and 81.6%, 97.4%, and 97.4% sensitivity for urinalysis, ultrasound, and ELISA, respectively (*p* < 0.001). The five positive dogs that were followed up after surgery showed a progressive decrease in mean absorbances in indirect ELISA (0.644, 0.516, 0.511, and 0.440, respectively). This study demonstrated that the indirect ELISA using the DE antigen could diagnose dioctophimosis regardless of the number, sex, and location of the parasites, with the potential to be used in epidemiological research and implementing immunological and molecular studies, opening new lines of research on *D. renale.*

## Resumo

*Dioctophyme renale* é um nematódeo que possui potencial zoonótico e acomete o rim de mamíferos carnívoros, silvestres e domésticos. Neste estudo busca-se avaliar a técnica de ELISA indireto frente metodologias de rotina utilizadas no diagnóstico da dioctofimose. Para isto, 38 cães participaram do estudo, sendo todos parasitados por *D. renale*, confirmados por cirurgia. Esses cães foram avaliados por ultrassom abdominal, urinálise e seus soros testado por ELISA indireto utilizando antígeno de excreção e secreção (DES) de adultos de *D. renale*. Destes, cinco cães foram acompanhados com coletas de soro, no dia zero (dia da cirurgia) e após 30, 60 e 90 dias da cirurgia para avaliar a cinética dos anticorpos. O ultrassom abdominal e ELISA indireto apresentaram sucesso no diagnóstico de 37 cães parasitados por *D. renale*, enquanto que a urinálise diagnosticou 29 animais. Os animais positivos possuíam de um a sete parasitos, 17 cães apresentaram infecções por macho e fêmeas, 15 somente por fêmeas e seis foram parasitados apenas por machos. Na avaliação da especificidade e sensibilidade, todas as técnicas apresentaram 100% de especificidade e 81,6%, 97,4%, 97,4% de sensibilidade para urinálise, ultrassom e ELISA, respectivamente (p < 0,001). Os cinco cães positivos que foram acompanhados após a cirurgia apresentaram diminuição progressiva nas médias de absorbâncias no ELISA indireto (0,644, 0,516, 0,511 e 0,440, respectivamente). O estudo demonstrou que o ELISA indireto utilizando o antígeno DES poderia diagnosticar dioctofimose, independentemente do número, sexo e localização dos parasitos, com potencial para ser utilizada em estudos epidemiológicos e na implementação de estudos imunológicos e moleculares, abrindo novas linhas de pesquisa sobre *Dioctophyme renale*.

## Introduction

*Dioctophyme renale* (Goeze, 1782) (Nematoda: Dioctophymatidae) parasitizes mainly the right kidney of domestic and wild mammals and can occasionally infect humans (Eiras et al., 2021; Measures, 2001). Although the kidney is the organ of choice, it can also be found in other locations, including the abdominal cavity and other urinary tract organs (Paras et al., 2018; Perera et al., 2021). Adult parasites in the pelvis of the kidney of their definitive hosts progressively destroy the cortical and medullary layer, reducing the kidney to a fibrous capsule. The contralateral kidney generally compensates for the loss of function of the parasitized kidney (Galiza et al., 2021; Measures, 2001). Dogs with dioctophimosis are usually asymptomatic, and when they present symptoms, they are not specific (Eiras et al., 2021).

*Dioctophyme renale* has a vast geographic distribution and has been reported in numerous American, European, and Asian countries (Eiras et al., 2021; Yang et al., 2019). This nematode has been documented throughout Brazil, with emphasis on the southern region of the country, where various studies have reported the adult parasite in dogs and cats (Brunner et al., 2022; Pedrassani et al., 2017; Perera et al., 2016; Rappeti et al., 2017), wild mammals (Pedrassani et al., 2017; Pesenti et al., 2012; Trindade et al., 2018), and paratenic hosts such as anurans (Pedrassani et al., 2009), fish (Mascarenhas et al., 2019), freshwater turtles (Mascarenhas & Müller, 2015; Mascarenhas et al., 2022) and snakes (Mascarenhas et al., 2018).

Domestic and wild mammals (definitive hosts) become infected by ingesting infective larva (L3) present in aquatic oligochaetes (intermediate hosts) or fish and anurans (paratenic hosts) (Mace & Anderson, 1975; Measures, 2001; Measures & Anderson, 1985). Thus, animal (and possibly human) infection is related to consuming water contaminated with the intermediate host and raw or undercooked meat from infected paratenic hosts (Measures, 2001; Yang et al., 2019).

Diagnosis can be performed by visually identifying the eggs in the urinary sediment, using imaging techniques that allow one to view the adult parasites, or by surgical and necropsy findings (Eiras et al., 2021; Measures, 2001). Among the techniques, urinary sediment analysis is one of the primary methods of diagnosing dioctophimosis in animals and humans (Ferreira et al., 2010; Pedrassani & Nascimento, 2015; Silveira et al., 2015; Yang et al., 2019). Nonetheless, this method becomes ineffective when renal parasitism occurs by immature females, *D. renale* males or when they are present in ectopic locations (Mesquita et al.; 2014; Pedrassani et al., 2015; Perera et al., 2021; Silveira et al., 2015).

New alternatives for diagnosing dioctophimosis have been developed to complement the existing methods for parasite detection and assist in parasite control. A recent study with excretion and secretion antigen (DES) obtained with adult *D. renale* cultures and using indirect ELISA for diagnosis showed 100 and 97.6% specificity and sensitivity, respectively (Capella et al., 2022). These are highly promising results for using this technique in the immunodiagnosis of dioctophimosis in dogs and to expand new lines of investigations on this nematode.

Thus, given the importance of this parasite, its zoonotic potential, and the difficulties in diagnosis, this study sought to evaluate the specificity and sensitivity of indirect ELISA using DES antigens and compare it with other techniques used in parasitological diagnosis.

## Materials and methods

### Dogs with dioctophimosis

We recruited 38 dogs from the Veterinary Hospital of the Universidade Federal de Pelotas (UFPel), all naturally infected with *D. renale* as confirmed by surgery. The procedures performed were approved by the ethics committee of UFPel (CEEA nos. 4395/15 and 57772/19). The dogs parasitized for *D. renale* were submitted to surgery, and the removed parasites were immediately sent to the laboratory to evaluate the number and sex of the parasites.

### Urine collection and analysis and abdominal ultrasound

Urine samples were collected by spontaneous urination; the egg presence was assessed using the centrifugal sedimentation method and observation under an optical microscope (Lopes et al., 2007). Abdominal ultrasound was performed using an ultrasound device with an 8C-RS (6-10 MHz) micro-convex multi-frequency transducer and a 12L-RS (7-12 MHz) linear multi-frequency transducer to analyze possible alterations in the abdominal organs and view the parasite.

### Serum sample collection and analysis

Serum samples were obtained by blood collection from the 38 parasitized dogs. Whole blood was collected by standard jugular venipuncture and drawn into sterile tubes without anticoagulant. The serum obtained were stored at -20 °C until use. To observe the antibody dynamics, five dogs underwent individual blood collections performed on the day of surgery to remove the parasites 0, 30, 60, and 90 days after surgery. The blood samples were collected from the jugular vein, and serum was obtained and stored at -20 °C until use.

### Indirect ELISA with DES antigens

The protocol used for antigen production and antibody detection against *D. renale* followed the method established by Capella et al. (2022). The indirect ELISA technique was performed using 1/400 dilution of serum, 1/25000 anti-dog IgG (whole molecule) — peroxidase antibody produced in rabbit (Sigma-Aldrich Cat. No. A 9042), and 1 µg/100 uL of antigen from excretion and secretion of the adult parasite with interplate controls. The cut-off point was determined as the mean absorbance of the negative control sera plus twice the standard deviation (0.374).

### Statistical analysis

The results of the ultrasound, urinalysis, and indirect ELISA techniques were analyzed using the MedCalc Ink 2019 software (version 18.9). The area under the curve (AUC), sensitivity and specificity of the different techniques to diagnose dioctophimosis in dogs were determined.

The mean absorbances of the positive dogs in the indirect ELISA were correlated with the number and sex of the parasites in the dogs. These results were submitted to analysis of variance, and the means were compared by Tukey’s test (*p* < 0.05) using the GraphPad-Prism software (version 7.0).

## Results

### Number and sex of the parasites collected per animal

The parasite load of the dogs was a minimum of one and a maximum of seven parasites. Of these, 15 dogs had only one parasite, 11 dogs had two parasites, five dogs had three parasites, three dogs had four parasites, three dogs had five parasites, and one dog had seven parasites. Regarding the sex of the parasites in the dogs, 17 showed mixed infections, 15 only females, and six only males.

### Indirect ELISA using DES

**Figure 1:**
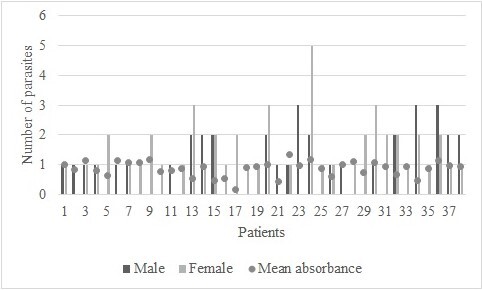
Number of male and female parasites obtained surgically and mean absorbance of sera from *Dioctophyme renale*-infected dogs in indirect ELISA using excretion and secretion antigen (DES).

In the indirect ELISA using the DES antigen, 37 dogs showed an absorbance value above the cut-off (0.374), which is considered positive for *D. renale*. The mean absorbance of the control sera from parasitized dogs was 0.871 ± 0.252. Regarding the number of parasites and their sex in the dogs, no association was observed between the absorbance in the indirect ELISA using the DES (Figure 1).

### Dogs followed up after surgery

**Figure 2:**
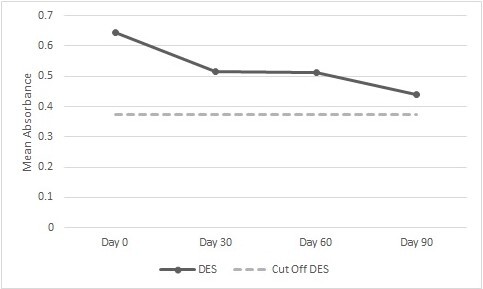
Mean absorbance in the indirect ELISA using the excretion and secretion antigen (DES) from five dogs parasitized by *Dioctophyme renale* on the day of parasite removal surgery (day 0) and 30 (day 30), 60 (day 60), and 90 days (day 90) after parasite removal surgery and a cut-off value of the DES antigens.

Dogs that were followed up 0, 30, 60, and 90 days after parasite removal surgery showed progressive decreases in mean absorbances in the indirect ELISA (0.644, 0.516, 0.511, and 0.440, respectively; Figure 2).

### Diagnosis of dioctophimosis by different techniques

The 38 patients (100%) tested positive for *D. renale* by surgery. However, abdominal ultrasound examination and indirect ELISA using DES antigen revealed that 37 patients (97.37%) were positive and one was negative (2.63%). In urine analysis, 29 (76.32%) tested positive and nine (23.68%) tested negative.

**Figure 3:**
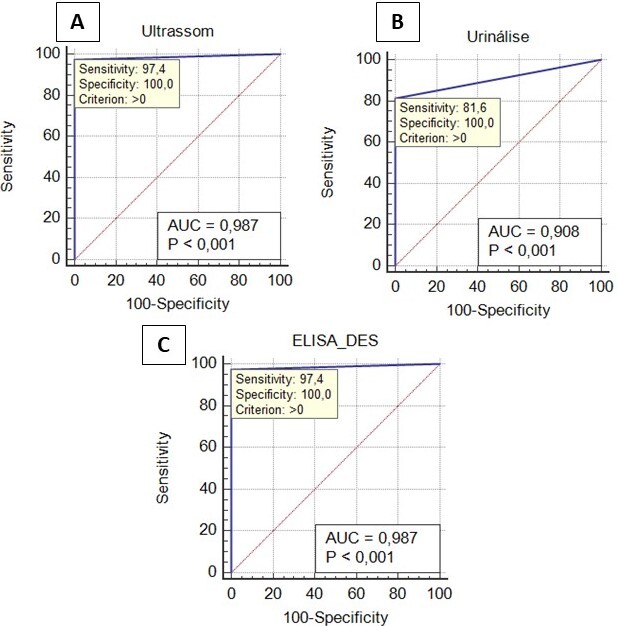
The area under the curve (AUC), specificity and sensitivity of different techniques used to diagnose *Dioctophyme renale*. A) abdominal ultrasound; B) urinalysis; C) indirect ELISA using excretory and secretory antigens (DES).

The AUC of the ultrasound, urinalysis, and indirect ELISA DES techniques were 0.987, 0.908, and 0.987, respectively (*p* < 0.001) in terms of specificity and sensitivity (Figure 3); all techniques presented 100% specificity. The ultrasound analysis and indirect ELISA showed 97.4% sensitivity and the urinalysis technique showed 81.6% sensitivity.

## Discussion

The cases of dioctophimosis are usually asymptomatic, and the adult nematode in the kidney is often diagnosed during necropsy or surgical procedures such as castration. Hence, it is vital to use more than one diagnostic technique to detect parasitosis before the host’s health is compromised. In addition, implementing serological techniques can assist in diagnosing young forms that may be migrating in different tissues of the definitive host (Eiras et al., 2021; Perera et al., 2021).

In the evaluation of 38 dogs, a single parasite was more frequent, which was also reported elsewhere. Kommers et al., (1999), Kano et al. (2003), Monteiro et al. (2002), Nakagawa et al., (2007), Pereira et al. (2006) and Rappeti et al. (2008) identified the presence of more than one *D. renale* in most dogs. When the maximum number of parasites was analyzed, it was seven, a result lower than the number observed by Monteiro et al. (2002) and Silveira et al. (2015), who reported up to 24 and 34 parasites per animal, respectively. As for the sex of the parasites in the *D. renale*-positive dogs, the infection by a single sex (female or male) was what predominated (55.26%), corroborating Pereira et al. (2006), who observed single infection in 50% of *D. renale* positive dogs. Nevertheless, Pedrassani et al. (2015) found a single infection in only 20% of *D. renale-*positive dogs; notably, in these studies, the number of dogs examined was smaller than in our study, which may have affected the results.

The number of parasites in the dogs that tested positive for *D. renale* showed no relationship with the mean absorbance in the indirect ELISA using the DES antigen, which has already been reported elsewhere with other helminths (Beraldi et al., 2008; Carmena et al., 2005). However, opposite results were observed by Fonseca et al. (2017) and Novák et al. (2017) when evaluating dose-dependent anti-*Toxocara canis* antibody levels in dogs, with small parasite loads resulting in lower antibody levels while high antibody levels were in animals with high infection rates. These differences may be associated with the sample of animals studied; in our study, the sample of dogs evaluated was made up of a heterogeneous group concerning relevant factors in the animals’ immune response, including nutritional status and age. The results obtained through experimental infections with control environment, food, and age have shown less variability in antibody levels in studies with *Echinococus granulosus* in dogs (Benito et al., 2001; Carmena et al., 2005).

In our study, in the analysis of the different techniques used to diagnose dioctophimosis, the urinalysis revealed that about 33% of dogs were false negatives, which corroborates previous studies that demonstrated and warned about the possibility of false negatives in cases involving infection only by males, immature females, or ectopic locations (Mesquita et al., 2014; Pedrassani et al., 2015; Silveira et al., 2015). It is emphasized that this technique has been used in various epidemiological studies of dioctophimosis in dogs (Colpo et al., 2007; Milanelo et al., 2009; Perera et al., 2016; Silveira et al., 2009), thereby making it possible for the presence of this parasite to be underestimated.

Some authors consider abdominal ultrasound a complementary and effective method to diagnose dioctophimosis (Caye et al., 2020; Radman et al., 2017; Silveira et al., 2015). Nonetheless, Rahal et al. (2014) compared different techniques routinely used to diagnose *D. renale* in dogs and reported that abdominal ultrasonography was ineffective in diagnosing all dogs with *D. renale*. This is quite similar to our findings, in which abdominal ultrasound examination did not lead to all dogs being diagnosed with dioctophimosis, which was confirmed by surgery.

In this study, the false negative dog on ultrasound was parasitized by a 17-cm male located in the abdominal cavity. We observed that the kidneys were preserved, and the cortical and medullary layers showed no changes, as well as the other organs. It is important to emphasize that the infective form (third-stage larva in the intermediate or paratenic host) penetrates the stomach wall after ingestion and passes through the liver and abdominal cavity, developing into the adult form that invades the kidney during this migration (Mace & Anderson, 1975). Hence, because the kidney did not show any changes, this parasite may have been migrating in the abdominal cavity and not yet penetrated the kidney. Greer et al. (2021) also observed no evidence of infection by ultrasound in a dog, and the presence of a female *D. renale* was confirmed in the right testicle during orchiectomy surgery.

When evaluating the results of the ELISA technique, we observed that the positive dogs at the time of surgery were also positive in the ELISA using the DES antigen except for one patient. This negative dog had two dead parasites inside the kidney in a state of decomposition, given the characteristics of the cuticle and staining. Although it was not possible to determine the period of parasite death, we found that, after surgical removal of the parasites, there was a gradual decrease in anti-DES antibody titers (0.640-0.440).

Additionally, this study showed that the ultrasound negative animal, whose parasite was migrating, tested positive in the ELISA, demonstrating that this technique can establish an early diagnosis before the parasite is located in the kidney and, consequently, decrease the aggravations caused by dioctophimosis. Thus, early diagnosis helps maintain the kidney parenchyma preserved, therefore not requiring the patient to undergo nephrectomy, maintaining the patient’s kidney preserved and resulting in higher quality and life expectancy (Radman et al., 2017).

Regarding the number and sex of the parasites in the *D. renale*-positive dogs, the mean absorbance in the ELISA showed no significant differences between dogs parasitized with a single parasite or infections by several parasites, even detecting a single young parasite migrating in the abdominal cavity.

## Conclusions

This study showed that the indirect ELISA technique using the DES antigen could diagnose dogs with *D. renale*, including parasites exclusively in the abdominal cavity, with 100% sensitivity and 97.4% specificity. Therefore, it is possible to suggest that the indirect ELISA technique for early diagnosis proved superior to ultrasound and urinalysis, with potential for use in research and clinical use, assisting in the control and prophylaxis of dioctophimosis. In addition, the immunodiagnostic technique may be a crucial tool in epidemiological, immunological, and molecular research to increase knowledge on the mechanisms involved in the parasite-host relationship, since it is an important parasite in animal health and a zoonosis.
